# The genome sequence of a caddisfly, *Limnephilus rhombicus* (Linnaeus, 1758)

**DOI:** 10.12688/wellcomeopenres.19331.1

**Published:** 2023-04-13

**Authors:** Gavin R. Broad, Benjamin W. Price, Ian Wallace, Natural History Museum Genome Acquisition Lab, Darwin Tree of Life Barcoding collective, Wellcome Sanger Institute Tree of Life programme, Wellcome Sanger Institute Scientific Operations: DNA Pipelines collective, Tree of Life Core Informatics collective, Darwin Tree of Life Consortium

**Affiliations:** 1 Natural History Museum, London, England, UK; 2 British Caddis Recording Scheme, Wirral, England, UK

**Keywords:** Limnephilus rhombicus, caddisfly, genome sequence, chromosomal, Trichoptera

## Abstract

We present a genome assembly from an individual male *Limnephilus rhombicus* (a caddisfly; Arthropoda; Insecta; Trichoptera; Limnephilidae). The genome sequence is 1,578.8 megabases in span. Most of the assembly is scaffolded into 30 chromosomal pseudomolecules, including the Z sex chromosome. The mitochondrial genome has also been assembled and is 21.9 kilobases in length. Gene annotation of this assembly on Ensembl identified 12,969 protein coding genes.

## Species taxonomy

Eukaryota; Metazoa; Ecdysozoa; Arthropoda; Hexapoda; Insecta; Pterygota; Neoptera; Endopterygota; Trichoptera; Integripalpia; Plenitentoria; Limnephiloidea; Limnephilidae; Limnephilinae; Limnephilini; *Limnephilus*; *Limnephilus rhombicus* (Linnaeus, 1758) (NCBI:txid692089).

## Background

*Limnephilus rhombicus* is a Holarctic species of caddisfly which is widespread and fairly common in Britain ( GBIF Secretariat, 2022). Many *Limnephilus* species have a large pale rhomboid mark in the centre of the wing, but this mark is especially large in this species, which probably encouraged Linnaeus to give it the specific name of *rhombicus*. The larva is striking, with a bulky case made of small pieces of plant material arranged at right angles to the axis. It has a strongly banded head and the anterior edge of the pronotum has a black stripe. Unfortunately, another four species have larvae that are so similar that microscopic examination is required to distinguish them from one another. The adult is distinctive and easily recognised using ( Wallace *et al.,* 2022). The larvae can only be identified to species when dead or anaesthetised using the key in Wallace, Wallace and Philipson (2003, which only works for final and penultimate instars; there is no key to identify small larvae, pupae or eggs.

The bulky case means the larva cannot exist in fast-flowing water, but, of the five species of this group, *L. rhombicus* is the one most likely to be found in slow-flowing rivers. In Britain, *L. rhombicus* seems not to be a species of water bodies that dry up over summer, but can be that elsewhere. *Limnephilus rhombicus* larvae are also found in larval form on lake shores and in canals, large ponds and fens, but as an adult it can be taken elsewhere and in light traps ( Wallace *et al.,* 2022).

The life cycle is not fully understood. The adults emerge in late spring with the females having undeveloped ovaries and this ovarian diapause seems to be under day-length control with them not maturing, mating and laying until late summer. The resulting larvae grow over winter. However, the diapause may be as short as six weeks and these early layers may be responsible for the not infrequent half-grown larvae found in summer ( Hoopes & Kim, 1987).

The larva feeds on mainly on dead, dying, and living plant material but, if the opportunity arises, will scavenge animal material and even be predaceous on *e.g. Simulium* larvae and other caddis species. Its predators are noted as large dytiscid beetle larvae, aeshnid dragonfly nymphs and Bullhead fish.

The high-quality genome sequence described here is the first one reported for *L. rhombicus*, to our knowledge, and has been generated as part of the Darwin Tree of Life project. It will aid in understanding the biology, physiology and ecology of the species, in addition to providing a mechanism to distinguish egg masses and early larval instars from those of its relatives.

### Genome sequence report

**Figure 1.**
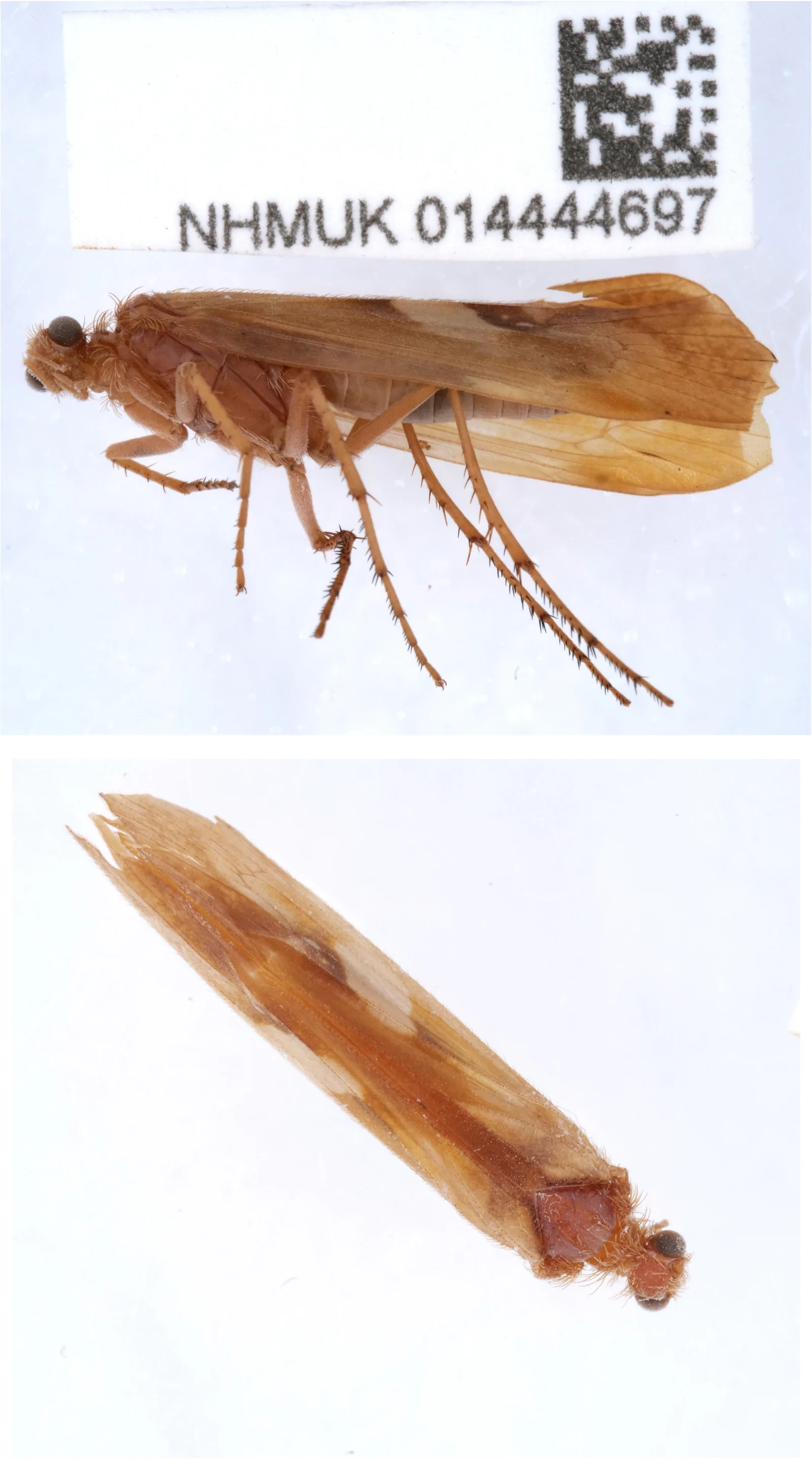
Photographs of the *Limnephilus rhombicus* (iiLimRhom1) specimen used for genome sequencing: (top) lateral view, (bottom) dorsal view.

The genome was sequenced from one male *L. rhombicus* specimen ( Figure 1) collected from Hever Castle, Kent (latitude 51.188367, longitude 0.119781). A total of 25-fold coverage in Pacific Biosciences single-molecule HiFi long reads and 40-fold coverage in 10X Genomics read clouds were generated. Primary assembly contigs were scaffolded with chromosome conformation Hi-C data. Manual assembly curation corrected 75 missing joins or mis-joins and removed two haplotypic duplications, reducing the scaffold number by 25.3%.

**Figure 2.**
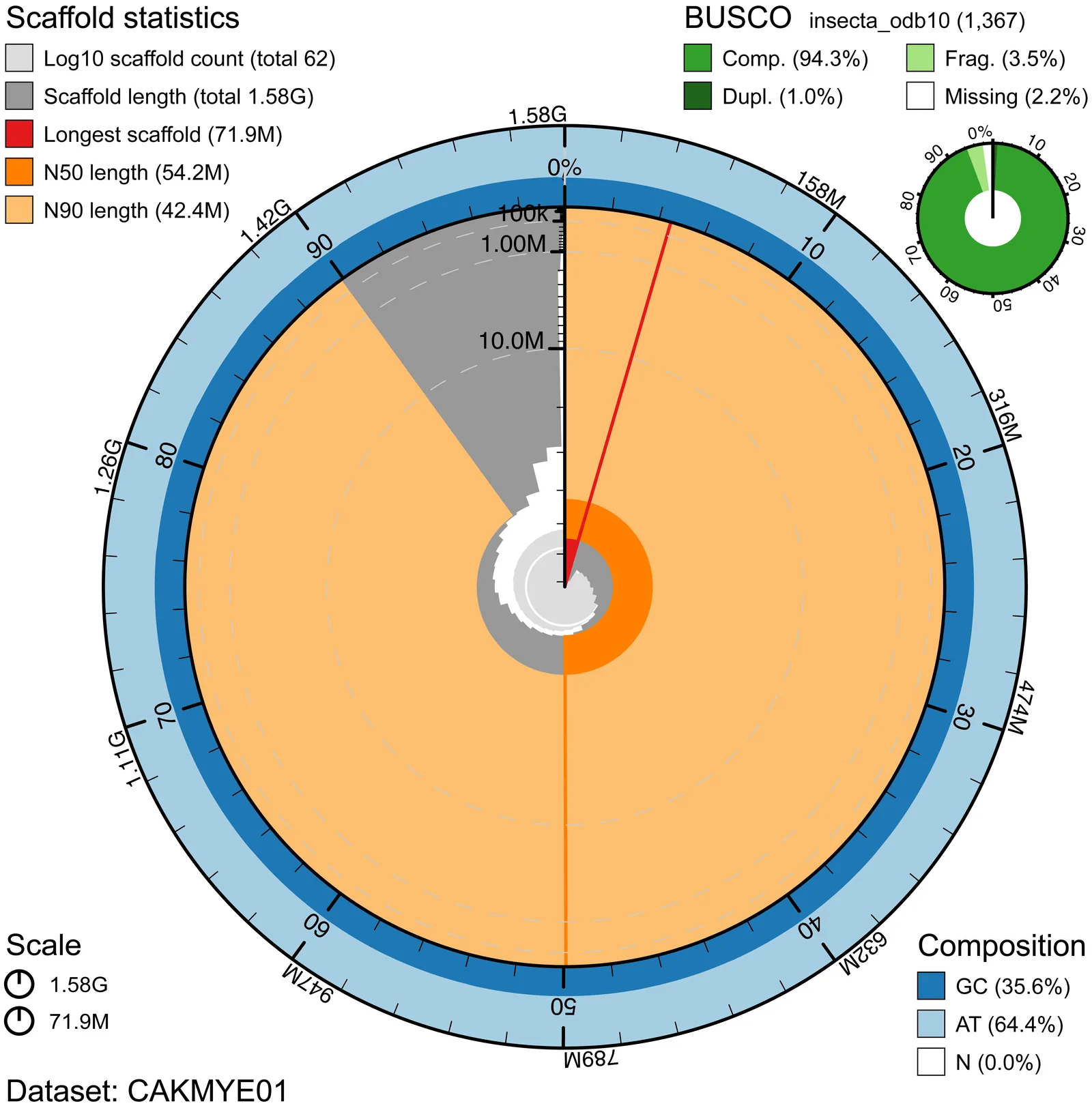
Genome assembly of *Limnephilus rhombicus*, iiLimRhom1.1: metrics. The BlobToolKit Snailplot shows N50 metrics and BUSCO gene completeness. The main plot is divided into 1,000 size-ordered bins around the circumference with each bin representing 0.1% of the 1,578,823,873 bp assembly. The distribution of scaffold lengths is shown in dark grey with the plot radius scaled to the longest scaffold present in the assembly (71,902,073 bp, shown in red). Orange and pale-orange arcs show the N50 and N90 scaffold lengths (54,234,467 and 42,438,354 bp), respectively. The pale grey spiral shows the cumulative scaffold count on a log scale with white scale lines showing successive orders of magnitude. The blue and pale-blue area around the outside of the plot shows the distribution of GC, AT and N percentages in the same bins as the inner plot. A summary of complete, fragmented, duplicated and missing BUSCO genes in the endopterygota_odb10 set is shown in the top right. An interactive version of this figure is available at https://blobtoolkit.genomehubs.org/view/iiLimRhom1.1/dataset/CAKMYE01/snail.

The final assembly has a total length of 1,578.8 Mb in 62 sequence scaffolds with a scaffold N50 of 54.2 Mb ( Table 1). Most (99.58%) of the assembly sequence was assigned to 30 chromosomal-level scaffolds, representing 29 autosomes, and the Z sex chromosome. Chromosome-scale scaffolds confirmed by the Hi-C data are named in order of size ( Figure 2– Figure 5; Table 2). While not fully phased, the assembly deposited is of one haplotype. Contigs corresponding to the second haplotype have also been deposited. The mitochondrial genome was also assembled and can be found as a contig within the multifasta file of the genome submission.

**Table 1.** Genome data for *Limnephilus rhombicus*, iiLimRhom1.2.

Project accession data		
Assembly identifier	iiLimRhom1.2	
Species	*Limnephilus rhombicus*	
Specimen	iiLimRhom1	
NCBI taxonomy ID	692089	
BioProject	PRJEB49540	
BioSample ID	SAMEA7849396	
Isolate information	iiLimRhom1, abdomen (PacBio and Chromium); head/thorax (Hi-C)	
Assembly metrics *		*Benchmark*
Consensus quality (QV)	50.2	*≥ 50*
*k*-mer completeness	99.96%	*≥ 95%*
BUSCO **	C:94.3%[S:93.3%,D:1.0%], F:3.5%,M:2.2%,n:1,367	*C ≥ 95%*
Percentage of assembly mapped to chromosomes	99.58%	*≥ 95%*
Sex chromosomes	Z chromosome	*localised homologous pairs*
Organelles	Mitochondrial genome assembled	*complete single alleles*
Raw data accessions		
PacificBiosciences SEQUEL II	ERR7979913, ERR7979914	
10X Genomics Illumina	ERR7783849–ERR7783852	
Hi-C Illumina	ERR7783853	
Genome assembly		
Assembly accession	GCA_929108145.2	
*Accession of alternate haplotype*	GCA_929113645.1	
Span (Mb)	1,578.8	
Number of contigs	271	
Contig N50 length (Mb)	10.8	
Number of scaffolds	61	
Scaffold N50 length (Mb)	54.2	
Longest scaffold (Mb)	71.9	
Genome annotation		
Number of protein-coding genes	12,969	
Number of non-coding genes	3,504	
Number of gene transcripts	22,728	

* Assembly metric benchmarks are adapted from column VGP-2020 of “Table 1: Proposed standards and metrics for defining genome assembly quality” from ( Rhie *et al.*, 2021). ** BUSCO scores based on the insecta_odb10 BUSCO set using v5.3.2. C = complete [S = single copy, D = duplicated], F = fragmented, M = missing, n = number of orthologues in comparison. A full set of BUSCO scores is available at https://blobtoolkit.genomehubs.org/view/iiLimRhom1.1/dataset/CAKMYE01/busco.

**Figure 3.**
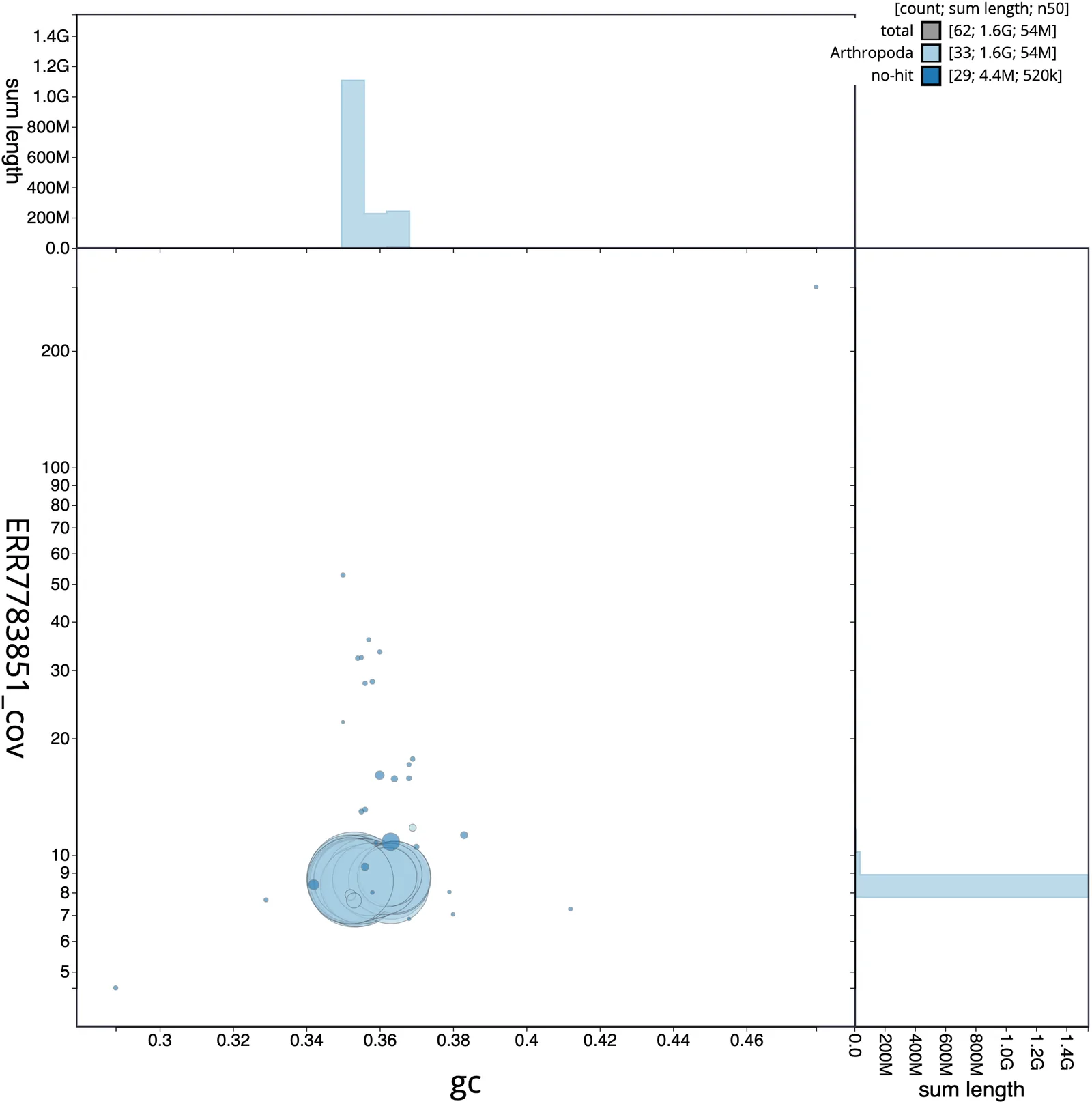
Genome assembly of *Limnephilus rhombicus*, iiLimRhom1.1: GC coverage. BlobToolKit GC-coverage plot. Scaffolds are coloured by phylum. Circles are sized in proportion to scaffold length. Histograms show the distribution of scaffold length sum along each axis. An interactive version of this figure is available at https://blobtoolkit.genomehubs.org/view/iiLimRhom1.1/dataset/CAKMYE01/blob.

**Figure 4.**
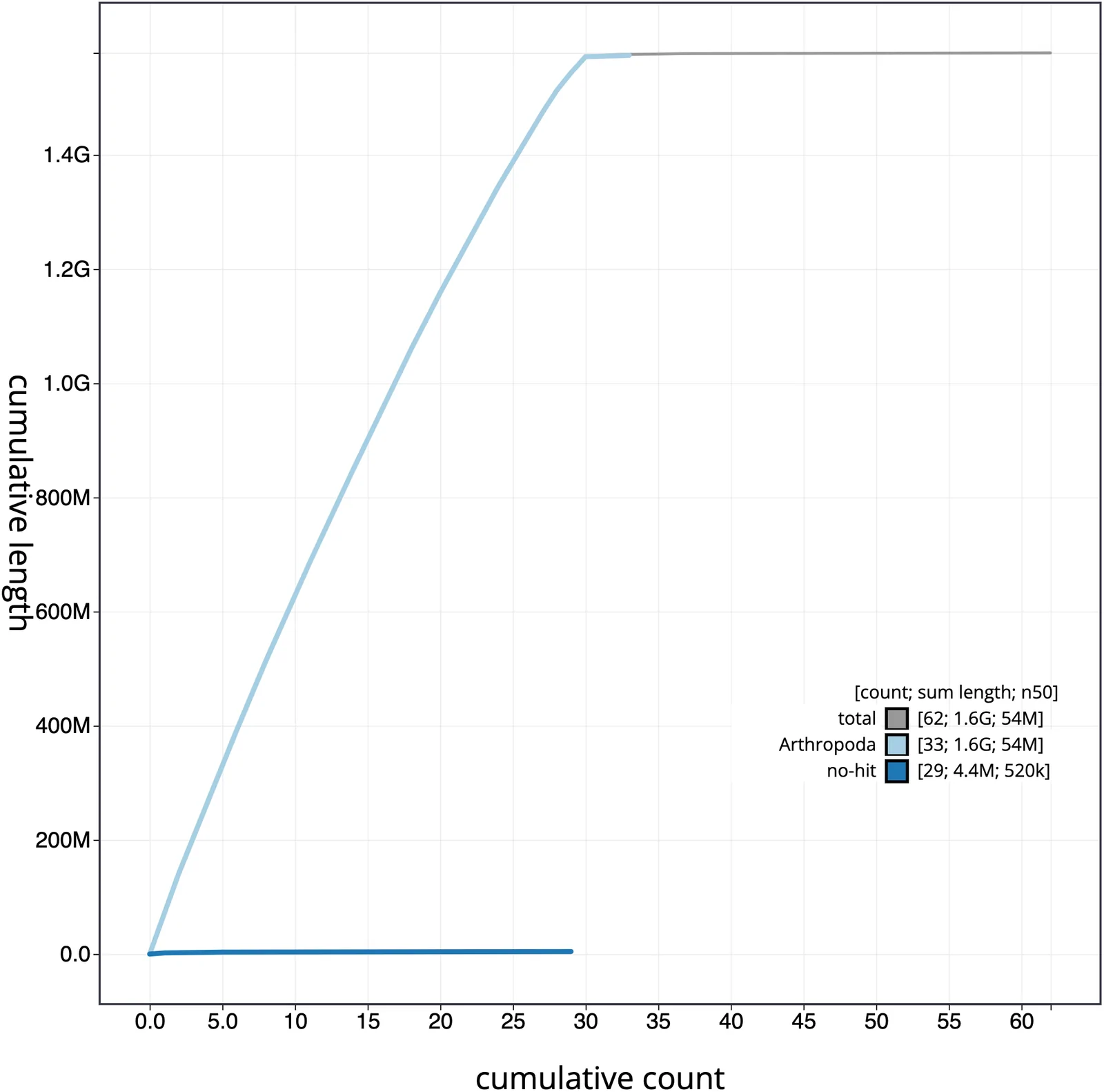
Genome assembly of *Limnephilus rhombicus*, iiLimRhom1.2: cumulative sequence. BlobToolKit cumulative sequence plot. The grey line shows cumulative length for all scaffolds. Coloured lines show cumulative lengths of scaffolds assigned to each phylum using the buscogenes taxrule. An interactive version of this figure is available at https://blobtoolkit.genomehubs.org/view/iiLimRhom1.1/dataset/CAKMYE01/cumulative.

**Figure 5.**
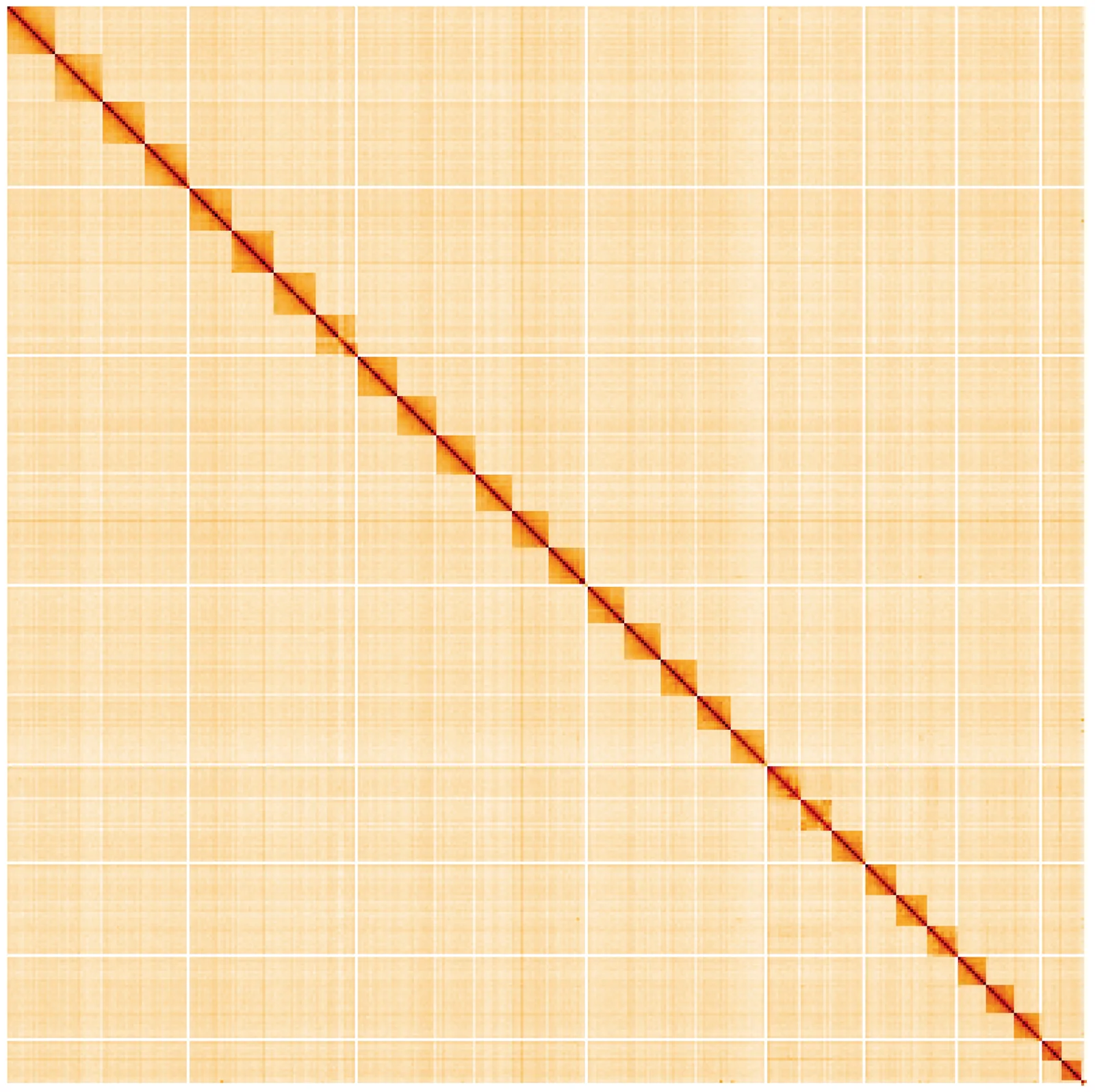
Genome assembly of *Limnephilus rhombicus*, iiLimRhom1.2: Hi-C contact map. Hi-C contact map of the iiLimRhom1.2 assembly, visualised using HiGlass. Chromosomes are shown in order of size from left to right and top to bottom. An interactive version of this figure may be viewed at https://genome-note-higlass.tol.sanger.ac.uk/l/?d=I5uw1QGuTP-YGeur3_nHzg.

**Table 2.** Chromosomal pseudomolecules in the genome assembly of *Limnephilus rhombicus*, iiLimRhom1.

INSDC accession	Chromosome	Size (Mb)	GC%
OV815270.1	1	71.9	35.3
OV815271.1	2	68.92	35.3
OV815272.1	3	63.9	35.3
OV815273.1	4	63.43	35.3
OV815274.1	5	62.72	35.5
OV815275.1	6	62.23	35.2
OV815277.1	7	59.54	35.4
OV815278.1	8	57.85	35.2
OV815279.1	9	56.8	35.4
OV815280.1	10	56.54	35.5
OV815281.1	11	55.03	35.3
OV815282.1	12	54.23	35.5
OV815283.1	13	54.2	35.5
OV815284.1	14	53.72	35.2
OV815285.1	15	53.42	35.3
OV815286.1	16	52.51	35.3
OV815287.1	17	51.86	35.6
OV815288.1	18	50.8	35.5
OV815289.1	19	48.55	36.3
OV815290.1	20	47.51	36.3
OV815291.1	21	46.74	35.8
OV815292.1	22	46.45	35.8
OV815293.1	23	45.27	35.4
OV815294.1	24	43.48	36.4
OV815295.1	25	42.44	35.7
OV815296.1	26	41.97	36.4
OV815297.1	27	39.15	36.1
OV815298.1	28	31.63	36.3
OV815299.1	29	28.37	36.2
OV815276.1	Z	61.09	35.2
OV815300.2	MT	0.02	19.7

The estimated Quality Value (QV) of the final assembly is 50.2 with *k*-mer completeness of 99.96%, and the assembly has a BUSCO v5.3.2 ( Manni *et al.*, 2021) completeness of 89.8% (single = 88.6%, duplicated = 1.2%), using the endopterygota_odb10 reference set ( *n* = 2,124) and 94.3% using the insecta_odb10 reference set ( *n* = 1,367).

Metadata for specimens, spectral estimates, sequencing runs, contaminants and pre-curation assembly statistics can be found at https://links.tol.sanger.ac.uk/species/692089.

### Genome annotation report

The *Limnephilus rhombicus* genome assembly ( GCA_929108145.2) was annotated using the Ensembl rapid annotation pipeline ( Table 1). The resulting annotation includes 22,728 transcribed mRNAs from 12,969 protein-coding and 3,504 non-coding genes.

## Methods

### Sample acquisition and nucleic acid extraction

A male *L. rhombicus* (iiLimRhom1) was collected from Hever Castle, Kent, UK (latitude 51.188367, longitude 0.119781) on 27 August 2020. The specimen was collected using a light trap by Gavin Broad (Natural History Museum) and identified by Benjamin Price (Natural History Museum), then snap-frozen on dry ice and stored at –80°C before extraction.

DNA was extracted at the Tree of Life laboratory, Wellcome Sanger Institute (WSI). The iiLimRhom1 sample was weighed and dissected on dry ice with tissue set aside for Hi-C sequencing. Abdomen tissue was disrupted using a Nippi Powermasher fitted with a BioMasher pestle. High molecular weight (HMW) DNA was extracted using the Qiagen MagAttract HMW DNA extraction kit. Low molecular weight DNA was removed from a 20-ng aliquot of extracted DNA using 0.8X AMpure XP purification kit prior to 10X Chromium sequencing; a minimum of 50 ng DNA was submitted for 10X sequencing. HMW DNA was sheared into an average fragment size of 12–20 kb in a Megaruptor 3 system with speed setting 30. Sheared DNA was purified by solid-phase reversible immobilisation using AMPure PB beads with a 1.8X ratio of beads to sample to remove the shorter fragments and concentrate the DNA sample. The concentration of the sheared and purified DNA was assessed using a Nanodrop spectrophotometer and Qubit Fluorometer and Qubit dsDNA High Sensitivity Assay kit. Fragment size distribution was evaluated by running the sample on the FemtoPulse system.

### Sequencing

Pacific Biosciences HiFi circular consensus and 10X Genomics read cloud DNA sequencing libraries were constructed according to the manufacturers’ instructions. DNA sequencing was performed by the Scientific Operations core at the WSI on Pacific Biosciences SEQUEL II (HiFi) and Illumina NovaSeq 6000 (10X) instruments. Hi-C data were also generated from head and thorax tissue of iiLimRhom1 using the Arima v2 kit and sequenced on the Illumina NovaSeq 6000 instrument.

### Genome assembly

Assembly was carried out with Hifiasm ( Cheng *et al.*, 2021) and haplotypic duplication was identified and removed with purge_dups ( Guan *et al.*, 2020). One round of polishing was performed by aligning 10X Genomics read data to the assembly with Long Ranger ALIGN, calling variants with freebayes ( Garrison & Marth, 2012). The assembly was then scaffolded with Hi-C data ( Rao *et al.*, 2014) using YaHS ( Zhou *et al.*, 2023). The assembly was checked for contamination as described previously ( Howe *et al.*, 2021). Manual curation was performed using HiGlass ( Kerpedjiev *et al.*, 2018) and Pretext ( Harry, 2022). The mitochondrial genome was assembled using MitoHiFi ( Uliano-Silva *et al.*, 2022), which performed annotation using MitoFinder ( Allio *et al.*, 2020).

To evaluate the assembly, MerquryFK assembly spectrum plots were generated to estimate *k*-mer completeness and consensus quality (QV) ( Rhie *et al.*, 2020). A Hi-C contact map for the final assembly was produced using minimap2 ( Li, 2021) in the Cooler file format ( Abdennur & Mirny, 2020). The genome was analysed, and BUSCO scores ( Manni *et al.*, 2021; Simão *et al.*, 2015) were generated within the BlobToolKit environment ( Challis *et al.*, 2020). Table 3 contains a list of software tool versions used and sources.

**Table 3.** Software tools: versions and sources.

Software tool	Version	Source
BlobToolKit	4.0.7	https://github.com/blobtoolkit/blobtoolkit
BUSCO	5.3.2	https://gitlab.com/ezlab/busco
FreeBayes	1.3.1-17-gaa2ace8	https://github.com/freebayes/freebayes
Hifiasm	0.16.1-r375	https://github.com/chhylp123/hifiasm
HiGlass	1.11.6	https://github.com/higlass/higlass
Long Ranger ALIGN	2.2.2	https://support.10xgenomics.com/genome-exome/software/pipelines/latest/advanced/other-pipelines
Merqury	MerquryFK	https://github.com/thegenemyers/MERQURY.FK
MitoHiFi	2	https://github.com/marcelauliano/MitoHiFi
PretextView	0.2	https://github.com/wtsi-hpag/PretextView
purge_dups	1.2.3	https://github.com/dfguan/purge_dups
YaHS	yahs-1.1.91eebc2	https://github.com/c-zhou/yahs

### Genome annotation

The Ensembl gene annotation system ( Aken *et al.*, 2016) was used to generate annotation for the *Limnephilus rhombicus* genome assembly (GCA_929108145.2: iiLimRhom1.2). Annotation was created primarily through alignment of transcriptomic data to the genome, with gap filling via protein to-genome alignments of a select set of proteins from UniProt ( UniProt Consortium, 2019).

### Ethics and compliance issues

The materials that have contributed to this genome note have been supplied by a Darwin Tree of Life Partner. The submission of materials by a Darwin Tree of Life Partner is subject to the Darwin Tree of Life Project Sampling Code of Practice. By agreeing with and signing up to the Sampling Code of Practice, the Darwin Tree of Life Partner agrees they will meet the legal and ethical requirements and standards set out within this document in respect of all samples acquired for, and supplied to, the Darwin Tree of Life Project. All efforts are undertaken to minimise the suffering of animals used for sequencing. Each transfer of samples is further undertaken according to a Research Collaboration Agreement or Material Transfer Agreement entered into by the Darwin Tree of Life Partner, Genome Research Limited (operating as the Wellcome Sanger Institute), and in some circumstances other Darwin Tree of Life collaborators.

## Data Availability

European Nucleotide Archive: *Limnephilus rhombicus*. Accession number PRJEB49540; https://identifiers.org/ena.embl/PRJEB49540 ( Wellcome Sanger Institute, 2021) The genome sequence is released openly for reuse. The *Limnephilus rhombicus* genome sequencing initiative is part of the Darwin Tree of Life (DToL) project. All raw sequence data and the assembly have been deposited in INSDC databases. Raw data and assembly accession identifiers are reported in Table 1.

## Author information

Members of the Natural History Museum Genome Acquisition Lab are listed here: https://doi.org/10.5281/zenodo.4790043.

Members of the Darwin Tree of Life Barcoding collective are listed here: https://doi.org/10.5281/zenodo.4893703.

Members of the Wellcome Sanger Institute Tree of Life programme are listed here: https://doi.org/10.5281/zenodo.4783585.

Members of Wellcome Sanger Institute Scientific Operations: DNA Pipelines collective are listed here: https://doi.org/10.5281/zenodo.4790455.

Members of the Tree of Life Core Informatics collective are listed here: https://doi.org/10.5281/zenodo.5013541.

Members of the Darwin Tree of Life Consortium are listed here: https://doi.org/10.5281/zenodo.4783558.
