# Supplementary material for: The genome sequence of a caddisfly, Limnephilus rhombicus (Linnaeus, 1758)
Source: Wellcome Open Res. 2023 Apr 13;8:170. [Version 1] doi: 10.12688/wellcomeopenres.19331.1 (PMC13550898; doi:10.12688/wellcomeopenres.19331.1)
Supplement: Peer review history

# Reviewer report: Reviewer response for version 1

**DOI:** 10.21956/wellcomeopenres.21416.r75198

**Published:** 2024-03-14

**Authors:** Sophie Tandonnet

**Affiliations:** 1 Universitat de Barcelona, Barcelona, Catalonia, Spain

**Editorial note: this peer-review report was updated on 22nd April 2024 at the request of the reviewer, as the reviewer noticed that some parts of the reviewer's original report draft were missing from the published online version.**

 As far as I can tell, this is a high quality genome report with adequate level of methods detail (except for the RNA-seq processing and Hi-C sample acquisition). The crucial missing information for me is about the Z chromosome. Specifically, there are missing references to the genetic sex determination of L. rhombicus, especially the presence of a Z chromosome. On that note, I believe that explaining how the Z chromosome was assigned its sex chromosome identity needs to be added.

**Background:**

 My main concern about the background section is that some sentences seem a bit broken. For example:

 - "The larvae can only be identified to species when dead or anaesthetised using the key in Wallace, Wallace and Philipson (2003 , which only works for final and penultimate instars; there is no key to identify small larvae, pupae or eggs."

 would read better as (modifications are in bold):

 "The larvae can only be identified **at species level** when dead or anaesthetised using the key in Wallace, Wallace and Philipson (2003 **)**, which only works for final and penultimate instars **(**there is no key to identify small larvae, pupae or eggs **)**."

 - "In Britain, *L. rhombicus* seems not to be a species of water bodies that dry up over summer, but can be that elsewhere.": I'm not quite sure I understand. Do the authors mean:

 "In Britain, *L. rhombicus* is usually not found in water bodies that dry up over summer, but this is still to determine elsewhere"?

 - " *Limnephilus rhombicus* larvae are also found in larval form on lake shores and in canals, large ponds and fens, but as an adult it can be taken elsewhere and in light traps ( Wallace *et al.,* 2022 )."

 could be changed to " *Limnephilus rhombicus* larvae **can also be** found on lake shores **, **in canals, large ponds and fens. **As** an adult, it can be **found** elsewhere and **captured using** light traps ( Wallace *et al.,* 2022 )."

 - "The larva feeds on mainly on dead, dying, and living plant material but, if the opportunity arises, will scavenge animal material and even be predaceous on *e.g. Simulium* larvae and other caddis species."

 should be changed to:

 "The larva **feeds mainly** on dead, dying, and living plant material but, if the opportunity arises, will scavenge animal material and even be **predatory** on *e.g. Simulium* larvae and other caddis species."

 - "in addition to providing a mechanism to distinguish egg masses and early larval instars from those of its relatives." This should be made more precise (as a mechanism for species determination is actually not explicitly laid out in the note):

 " **in addition to providing valuable resources to ascertain the species identity in egg masses and early larval instars**"

 Minor comment: the text states that the larva is striking and it made me curious to have a picture of it. Could one be included?

**Genome Sequence report:**

 "The final assembly has a total length of 1,578.8 Mb" -> I think the authors meant 1.578.8 Gb (not Mb).

 The links in the legends of figures 2, 3 and 4 did not work for me.

 Figure 5: Would it be possible to add the chromosome names to this figure? Which chromosome is the 8th chromosome (in Fig 5)? It has a sort of "split" pattern. Do you know to what this is due?

 "The estimated Quality Value (QV) ... is 50.2 ": could the authors give the reader a sense of what the QV of 50 represents? Is 50 a good score? What would be the perfect score?

**Genome Annotation report:**

 I really liked seeing that there is an annotation available for the genome. One suggestion: I would be nice to have a BUSCO score of the predicted proteome.

**Methods:**

**Sample acquisition and nucleic acid extraction:**

 In this section, please add the details of how the Hi-C sample was acquired (Covaris cryoPREP Automated Dry Pulveriser?). 

 Also add how the RNA extraction was performed and which samples/stages were used for gathering the transcriptomic data.

**Sequencing:**

 Add detail of how the RNA samples were sequenced.

 Also add a section of how the RNA-seq data was processed (programs, parameters, etc).

**Genome assembly:**

 The authors cite all the programs and versions used. Were all the parameters left by default? If some parameters were changed from the programs' default please add these details.

 Table 3: Minimap2 missing

**Data availability:**

 I could find the Pacbio, 10x data and Hi-C data here: https://www.ebi.ac.uk/ena/browser/view/PRJEB49538

 However I could not find the RNA-seq/transcriptomic data although the previous web page states:

 "This project collects the genomic and transcriptomic data generated for Limnephilus rhombicus [...]"

 Could the authors add the transcriptomic data or reference where it can be downloaded? This information could also be added to Table 1.

Are sufficient details of methods and materials provided to allow replication by others?

Partly

Is the rationale for creating the dataset(s) clearly described?

Yes

Are the datasets clearly presented in a useable and accessible format?

Partly

Are the protocols appropriate and is the work technically sound?

Yes

Reviewer Expertise:

Bioinformatics (genomics and transcriptomics), genetics, evolutionary biology

I confirm that I have read this submission and believe that I have an appropriate level of expertise to confirm that it is of an acceptable scientific standard, however I have significant reservations, as outlined above.

# Reviewer report: Reviewer response for version 1

**DOI:** 10.21956/wellcomeopenres.21416.r66932

**Published:** 2023-10-10

**Authors:** Pavel B Klimov

**Affiliations:** 1 Purdue University, West Lafayette, Indiana, USA

This an impressive  chromosome-level genomic assembly. However, I have a few comments:

 2) Are the protocols appropriate and is the work technically sound? I scored "Partly" because if the following:

 2.1) The manuscript inconsistently refers to two different assembly versions, iiLimRhom1.1 and iiLimRhom1.2. The key metrics (length, BUSCO) are different for these assemblies. To avoid confusion, I suggest that the ms is based on a single assembly version (or if citing the two assemblies is necessary than they should be described separately).

 2.2) Genome annotation was described only cursory (~3 lines). To ensure reproducibility, I suggest that the actual command lines are given (or describe all the steps if this was done manually). Furthermore, these three lines indicate the utilization of transcriptomic data; however, the manuscript does not specify whether transcriptomic sequencing was conducted as part of this study or if a third-party dataset was employed.

 2.3) This assembly was visualized in BlobTools, a program is designed for metagenomic assembly decontamination and to visualize assembly contiguity based on scaffold/contigs. I believe that these diagrams are misleading because  this assembly is a chromosome-level assembly, not scaffold-level.

 3) Are sufficient details of methods and materials provided to allow replication by others? I scored "No" because of aspect 2.2

 4) Are the datasets clearly presented in a usable and accessible format? I scored "Partly" because of aspect 2.2. Also, the gene predictions were saved in GTF and GFF3 formats in the Ensembl page. However, the GenBank submission lacks any annotation. I suggest  that your gene predictions are deposited into NCBI GenBank to ensure the visibility of the data.

Are sufficient details of methods and materials provided to allow replication by others?

No

Is the rationale for creating the dataset(s) clearly described?

Yes

Are the datasets clearly presented in a useable and accessible format?

Partly

Are the protocols appropriate and is the work technically sound?

Partly

Reviewer Expertise:

Evolutionary Biology, Genomics.

I confirm that I have read this submission and believe that I have an appropriate level of expertise to confirm that it is of an acceptable scientific standard, however I have significant reservations, as outlined above.
